# Diagnostik und Therapie von Tumoren mit *NTRK*-Genfusionen

**DOI:** 10.1007/s00292-020-00864-y

**Published:** 2020-11-30

**Authors:** Albrecht Stenzinger, Cornelis M. van Tilburg, Ghazaleh Tabatabai, Florian Länger, Norbert Graf, Frank Griesinger, Lukas C. Heukamp, Michael Hummel, Thomas Klingebiel, Simone Hettmer, Christian Vokuhl, Sabine Merkelbach-Bruse, Friedrich Overkamp, Peter Reichardt, Monika Scheer, Wilko Weichert, C. Benedikt Westphalen, Carsten Bokemeyer, Philipp Ivanyi, Sonja Loges, Peter Schirmacher, Bernhard Wörmann, Stefan Bielack, Thomas T. W. Seufferlein

**Affiliations:** 1 Allgemeine Pathologie und pathologische Anatomie, Pathologisches Institut, Universitätsklinikum Heidelberg, Im Neuenheimer Feld 224, 69120 Heidelberg, Deutschland; 2 Hopp-Kindertumorzentrum Heidelberg (KiTZ), Deutsches Krebsforschungszentrum (DKFZ), Universitätsklinikum Heidelberg, Heidelberg, Deutschland; 3 Abteilung Neurologie mit interdisziplinärem Schwerpunkt Neuroonkologie, Universitätsklinikum Tübingen und Hertie-Institut für Klinische Hirnforschung, Eberhard Karls Universität Tübingen, Tübingen, Deutschland; 4 Institut für Pathologie, Medizinische Hochschule Hannover, Hannover, Deutschland; 5 Klinik für Pädiatrische Onkologie und Hämatologie, Universitätsklinikum des Saarlandes, Medizinische Fakultät, Universität des Saarlandes, Homburg, Deutschland; 6 Klinik für Hämatologie und Onkologie, Universitätsklinik für Innere Medizin – Onkologie, Pius-Hospital Oldenburg, Oldenburg, Deutschland; 7 Institut für Hämatopathologie Hamburg, Hamburg, Deutschland; 8 Institut für Pathologie (CCM), Charité – Universitätsmedizin Berlin, Berlin, Deutschland; 9 Klinik für Kinder- und Jugendmedizin, Universitätsklinikum Frankfurt, Frankfurt, Deutschland; 10 Klinik für Pädiatrische Hämatologie und Onkologie, Zentrum für Kinder- und Jugendmedizin, Universitätsklinikum Freiburg, Freiburg, Deutschland; 11 Sektion Kinderpathologie, Institut für Pathologie, Universitätsklinikum Bonn, Bonn, Deutschland; 12 Institut für Allgemeine Pathologie und Pathologische Anatomie, Uniklinik Köln, Köln, Deutschland; 13 OncoConsult Overkamp GmbH, Berlin, Deutschland; 14 Onkologie und Palliativmedizin, Helios Klinikum Berlin-Buch, Berlin, Deutschland; 15 Pädiatrie 5 – Onkologie, Hämatologie und Immunologie, Zentrum für Kinder‑, Jugend- und Frauenmedizin – Olgahospital, Stuttgart Cancer Center, Klinikum Stuttgart, Stuttgart, Deutschland; 16 Institut für Allgemeine Pathologie und Pathologische Anatomie, Technische Universität München, München, Deutschland; 17 Medizinische Klinik und Poliklinik III, Klinikum der Universität München, Ludwig-Maximilians-Universität München, München, Deutschland; 18 Zentrum für Onkologie, II. Medizinische Klinik und Poliklinik (Onkologie, Hämatologie, Knochenmarktransplantation mit Abteilung für Pneumologie), Universitätsklinikum Hamburg-Eppendorf, Hamburg, Deutschland; 19 Klinik für Hämatologie, Hämostaseologie, Onkologie und Stammzelltransplantation, Medizinische Hochschule Hannover, Hannover, Deutschland; 20 Zentrum für experimentelle Medizin, Institut für Tumorbiologie, Universitätsklinikum Hamburg- Eppendorf, Hamburg, Deutschland; 21 Medizinische Klinik mit Schwerpunkt Hämatologie, Onkologie und Tumorimmunologie (CVK), Charité – Universitätsmedizin Berlin, Berlin, Deutschland; 22 Klinik für Innere Medizin I, Universitätsklinikum Ulm, Ulm, Deutschland; 23 Abteilung für Personalisierte Medizinische Onkologie, Deutsches Krebsforschungszentrum (DKFZ), Heidelberg, Deutschland; 24 Universitätsklinikum Mannheim, Mannheim, Deutschland

**Keywords:** NTRK, Genfusion, Translokation, Larotrectinib, Entrectinib, NTRK, Gene fusion, Translocation, Larotrectinib, Entrectinib

## Abstract

*NTRK*-Genfusionen sind seltene genetische Alterationen, die tumorentitätenübergreifend vorkommen können. Während sie in den meisten soliden Tumoren nur sehr niederfrequent vorkommen, lassen sie sich in bestimmten Tumoren wie dem infantilen Fibrosarkom, dem kongenitalen mesoblastischen Nephrom und dem sekretorischen Mamma- oder Speicheldrüsenkarzinom jedoch häufig nachweisen. *NTRK*-Genfusionen bzw. TRK-Fusionsproteine gelten als starke onkogene Treiber. Bei Nachweis von *NTRK*-Genfusionen können TRK-Inhibitoren unabhängig von der Tumorentität eingesetzt werden. Vertreter sind Entrectinib und Larotrectinib. Bislang ist nur Larotrectinib in der Europäischen Union zugelassen. Für beide wurden Wirksamkeit und Verträglichkeit in Phase-I- und Phase-II-Studien gezeigt. Die Seltenheit der TRK-Fusionstumoren stellt diagnostische und klinische Prozesse vor große Herausforderungen: Einerseits sollen alle Patienten mit TRK-Fusionstumoren identifiziert werden, andererseits sind epidemiologische und histologische Aspekte sowie Ressourcen zu berücksichtigen. Basierend auf diesen Punkten möchten wir einen Diagnosealgorithmus für TRK-Fusionstumoren vorschlagen, außerdem stellen wir aktuelle Daten zu den TRK-Inhibitoren vor.

**Zusatzmaterial online:**

Die Online-Version dieses Beitrags (10.1007/s00292-020-00864-y) enthält weitere Quellen, ergänzende Inhalte sowie Tabellen zu Larotrectinib und Entrectinib [Verträglichkeit; Baseline-Charakteristika; Wirksamkeit von Entrectinib bei TRK-Fusionstumoren; Therapiebedingte unerwünschte Ereignisse]. Beitrag und Zusatzmaterial stehen Ihnen auf www.springermedizin.de zur Verfügung. Bitte geben Sie dort den Beitragstitel in die Suche ein, das Zusatzmaterial finden Sie beim Beitrag unter „Ergänzende Inhalte“.

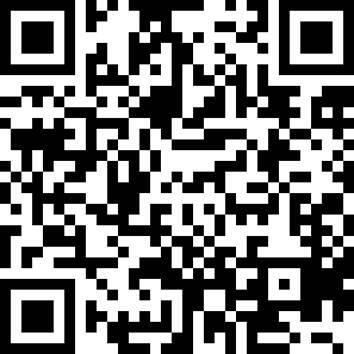

## Abstract (en)

*NTRK* gene fusions are sporadic genetic alterations that can occur across tumor entities. Whereas they are quite rare in most solid tumors they are present at much higher frequencies in certain rare tumors such as infantile fibrosarcoma, congenital mesoblastic nephroma, secretory breast, or salivary gland carcinoma. *NTRK* gene fusions or TRK fusion proteins are considered strong oncogenic drivers. If *NTRK* gene fusions are detected, TRK inhibitors such as entrectinib and larotrectinib can be used regardless of the tumor entity. So far only larotrectinib is approved in the European Union. Both drugs have been shown to be effective and well tolerated in phase I and phase II studies. The low prevalence of TRK fusion-positive cancers poses challenges for diagnostic and clinical work-flows. On one hand, patients with *NTRK *gene fusions should be identified; on the other hand, epidemiological, histological, and resource-related aspects have to be taken into account. Based on these premises, we suggest a diagnostic algorithm for TRK fusion cancers and present current data on TRK inhibitors.

Diagnostik und Therapie vieler Krebserkrankungen haben sich in den letzten Jahren enorm gewandelt. So stehen zunehmend molekular zielgerichtete Therapien und/oder Immuntherapien zur Verfügung, die passgenau zu molekulargenetischen und -pathologischen Veränderungen bei einer bestimmten Tumorerkrankung eingesetzt werden können. Dies hat den Stellenwert der molekularpathologischen und -genetischen Diagnostik nachhaltig verändert. Jetzt zeichnet sich ein weiterer Schritt ab: eine tumorentitätenübergreifende, möglicherweise sogar „tumoragnostische“ Therapiestrategie.

Eines der ersten Beispiele für dieses Konzept sind Inhibitoren der Tropomyosin-Rezeptor-Kinasen (TRK). Deren Einsatz richtet sich nicht primär nach der Tumorentität, sondern nach dem Nachweis der zugrunde liegenden molekularen Veränderung, der *NTRK*-Genfusion. Tumoren mit *NTRK*-Genfusionen sind selten. Dies stellt für die klinischen Prozesse eine große Herausforderung dar: Welcher Patient soll wann und wie von wem getestet werden und wann sollte die Therapie mit einem TRK-Inhibitor begonnen werden? Im Folgenden möchten wir den Hintergrund aktueller Studiendaten sowie einen möglichen Diagnosealgorithmus für TRK-Fusionstumoren vorschlagen und erläutern.

## Die TRK-Familie

Bei den TRK handelt es sich um Rezeptortyrosinkinasen, die physiologisch vorwiegend im menschlichen Nervengewebe exprimiert werden [46]. Diese Transmembranrezeptorfamilie hat 3 Mitglieder: TRKA, TRKB und TRKC [10, 32]. Liganden aller 3 Rezeptoren sind Neurotrophine, die mit hoher Affinität binden [10, 32]. Durch die Ligandenbindung kommt es zu Homodimerisierung (und möglicherweise auch Heterodimerisierung) der transzellulären Rezeptor-Tyrosinkinase mit konsekutiver Aktivierung der Tyrosinkinase und subsequenter Aktivierung verschiedener intrazellulärer Signalwege [10]. Dieses Signal steuert u. a. die neuronale Entwicklung und Differenzierung sowie physiologische neuronale Prozesse [10, 15, 32]: TRKA spielt für Proliferation und Differenzierung von Zellen eine Rolle [2, 59], TRKB-induzierte Signalwege unterstützen die neuronale Differenzierung und das Überleben der Zellen [2, 59]. TRKC ist in Prozesse eingebunden, die die Apoptose verhindern und die Motilität von Zellen fördern [2]. Zusammenfassend sind TRK über verschiedene Signalwege an der Regulation der Motorik, Propriozeption, Schmerzempfindung, Thermoregulation, Gedächtnisleistung, Stimmung, Appetit und Körpergewicht beteiligt [15].

## *NTRK*-Genfusionen als onkogene Treiber

Die für TRKA, TRKB und TRKC kodierenden Gene sind *NTRK1, NTRK2* und *NTRK3* (NTRK = neurotrophe Tyrosin-Rezeptor-Kinase) [10, 32]. Die häufigste Ursache für eine onkogene TRK-Aktivierung ist das Vorliegen von *NTRK*-Genfusionen [10, 32]. Ihnen liegen intra- oder interchromosomale Translokationen zugrunde, bei denen das 3′-Ende von *NTRK1, NTRK2 *oder *NTRK3, *das die codierenden Abschnitte für die Kinasedomäne enthält, mit dem 5′-Ende verschiedener Gene (i. d. R. aus derselben funktionellen Klasse) [71] verbunden wird, die i. d. R. Dimerisierungsdomänen enthalten. Das daraus translatierte Fusionsprotein (chimäres Onkoprotein) verursacht eine konstitutive (dauerhafte), ligandenunabhängige Aktivierung der TRK-Kinasedomäne [10, 32]. Die dauerhafte Aktivierung der TRK führt zur Aktivierung eines Signalnetzwerks, das u. a. MAP-Kinase, Proteinkinase C (PKC), die Phosphatidylinositol-3-Kinase (PI3K) und AKT umfasst und zur Stimulation von Proliferation bzw. Hemmung von Apoptosesignalen führt [2, 42, 46].

Auf genetischer Ebene sind neben häufigen Fusionsgenen (z. B. *ETV6-NTRK3*) zahlreiche Varianten mit niedriger Prävalenz zu beobachten. Bisher wurden nicht nur über 80 Genfusionspartner identifiziert, sondern auch unterschiedliche, in die Fusion involvierte Exone der Fusionspartner (unterschiedliche Bruchpunkte), sodass die Gesamtzahl aller Varianten weitaus höher liegt [47, 61, 72]. Es ist wichtig festzuhalten, dass sich dieses Bild fortlaufend ändern wird, da anzunehmen ist, dass mit steigender Testrate weitere Fusionsvarianten identifiziert werden.

*NTRK*-Genfusionen oder TRK-Fusionsproteine sind starke onkogene Treiber bei unterschiedlichen Krebserkrankungen [46]. Das gleichzeitige Auftreten einer *NTRK*-Genfusion mit anderen starken onkogenen Treibern ist nach derzeitigem Stand der Literatur im TRK-Inhibitor-naiven Setting selten (Konzept der „mutual exclusivity“ [51]). In Verbindung mit einer spezifischen Morphologie kann der Nachweis einer *NTRK*-Genfusion die Diagnose unterstützen, z. B. bei sekretorischen Karzinomen der Speicheldrüse oder infantilen Fibrosarkomen (IFS). Zukünftige Studien und Forschungsprojekte sind jedoch notwendig, um zu klären, in welcher Art und Weise und in welchem Umfang der tumorbiologische Kontext, in den die Genfusion eingebettet ist und der durch die Histologie (Tumorentität) angezeigt wird, die onkogene Potenz von *NTRK*-Genfusionen moduliert und möglicherweise das Therapieansprechen konventioneller wie auch zielgerichteter Therapieansätze bzw. auch die Krankheitsprognose beeinflusst. Auch die Frage, inwieweit *NTRK*-Fusionsvarianten die Tumorbiologie und das Therapieansprechen modulieren, ist an Hand der bisherigen Studiendaten nicht abschließend zu klären und bedarf weiterer wissenschaftlicher Untersuchungen. So legen beispielsweise mehrere Berichte zu ALK(anaplastische Lymphomkinase)-positiven Lungenkarzinomen nahe, dass Translokationen mit verschiedenen Fusionspartnern und Bruchpunkten unterschiedliche biologische Eigenschaften aufweisen [68] und mit unterschiedlichen klinischen Therapieverläufen assoziiert [8, 37] sein können. Bei genetisch komplexen Tumoren, nachgewiesen bei Osteosarkomen, können *NTRK*-Fusionen auch zu nichtfunktionalen Genprodukten führen [3].

Mögliche weitere Ursachen für eine onkogene TRK-Aktivierung sind *NTRK*-Genamplifikationen, die bei ca. 2,2 % der metastasierten Karzinome vorkommen können [36], und somatische *NTRK*-Punktmutationen, die die Kinasedomäne von TRKB betreffen, z. B. die *NTRK2*-Mutationen T695I und D751N, die im kolorektalen Karzinom beobachtet wurden [10]. Ferner konnten auch alternative *NTRK1*-Splicevarianten nachgewiesen werden wie TRKAIII oder ∆TRKA beim Neuroblastom bzw. bei der akuten myeloischen Leukämie (AML) [10]. Weiterhin ist eine TRK-Überexpression ohne Nachweis einer genetischen Alteration möglich, z. B. bei Tumoren der Brust, der Haut und der Lunge sowie beim Neuroblastom. Nach derzeitigem Kenntnisstand ist allerdings unklar, ob diese Veränderungen aus tumorbiologischer und therapeutischer Sicht als den *NTRK*-Genfusionen gleichwertig anzusehen sind. Zur Klärung dieser Fragestellung sind neben präklinischen Untersuchungen prospektive klinische Studien notwendig.

### Häufigkeit von *NTRK*-Genfusionen

**Figure 1:**
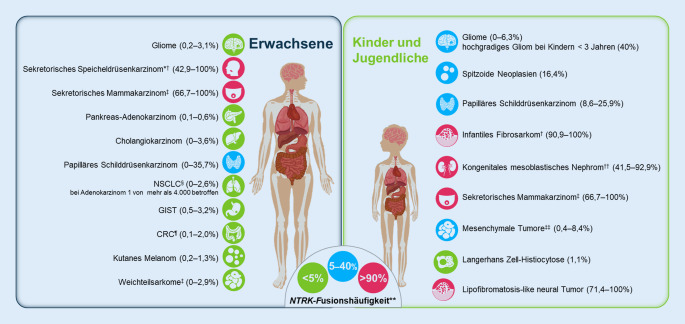

Insgesamt sind *NTRK*-Genfusionen selten und nur bei etwa 0,3–0,5 % aller soliden Tumoren nachweisbar ([44, 57]; Abb. 1). *NTRK*-Genfusionen finden sich auch bei hämatologischen Krebserkrankungen wie der AML und der akuten lymphatischen Leukämie (ALL) mit einer Prävalenz von maximal 0,1 % [60]. Okamura et al. untersuchten die Prävalenz von *NTRK*-Genfusionen in fast 13.500 Gewebeproben von Kindern, Heranwachsenden und Erwachsenen mit soliden Tumoren oder hämatologischen Krebserkrankungen [44]. Sie fanden eine Häufigkeit dieser Alterationen von 0,31 % (31 von 9966 Tumorgeweben) bei Erwachsenen und 0,34 % (12 von 3501 Tumorgeweben) bei Kindern und Heranwachsenden. Allerdings haben alle bisherigen Untersuchungen zur Häufigkeit von *NTRK*-Genfusionen Limitationen, da sie hinsichtlich Sensitivität und Spezifität mit sehr unterschiedlichen diagnostischen Methoden durchgeführt wurden und aufgrund der geringen Prävalenz und geringen untersuchten Fallzahlen je Tumorentität die Aussagekraft statistischer Analysen eingeschränkt ist. Dies gilt insbesondere bei Tumortypen mit vergleichsweise niedrigem Vorkommen von *NTRK*-Genfusionen [42, 44].

Interessanterweise lassen sich *NTRK*-Genfusionen bei bestimmten, seltenen Tumoren häufig nachweisen ([1, 10, 32]; Abb. 1). So ist z. B. die *ETV6-NTRK3*-Fusion mit einer Prävalenz von >90 % pathognomonisch bei Krebserkrankungen wie dem IFS, dem kongenitalen mesoblastischen Nephrom (CMN, zellulärer Subtyp), dem sekretorischen Mammakarzinom oder dem sekretorischen Speicheldrüsenkarzinom ([10, 64]; Abb. 1).

Die Analyse der derzeit verfügbaren Daten zeigt auf, dass im Gegensatz zu beispielsweise *ALK*-Genfusionen, die vorwiegend im nichtkleinzelligen Lungenkarzinom (NSCLC) [52], bei großzelligen Lymphomen [19] und in inflammatorischen myofibroblastischen Tumoren [4] beobachtet werden, die Fusionspartner von *NTRK* und die involvierten Exone der verschiedenen Gene vielfältiger sind, sodass eine genaue Kenntnis der verfügbaren Methoden zum Nachweis einer *NTRK*-Genfusion besonders wichtig ist [47].

## Diagnostik von *NTRK*-Genfusionen

### Untersuchungsmethoden

Die klinische Anwendung von TRK-Inhibitoren wie Entrectinib oder Larotrectinib setzt den erbrachten Nachweis einer *NTRK*-Genfusion voraus [5, 21]. In den verfügbaren Studien mit den TRK-Inhibitoren wurde keine sog. Companiondiagnostik evaluiert und festgelegt. Beispielsweise erfolgte in den klinischen Untersuchungen zur Analyse der Wirksamkeit und Sicherheit von Larotrectinib der Nachweis einer TRK-Fusion mittels Next Generation Sequencing (NGS), Fluoreszenz-in-situ-Hybridisierung (FISH) oder Reverse-Transkriptase-Polymerase-Kettenreaktion (RT-PCR) mit unterschiedlichen Plattformen in unterschiedlichen Laboren [5, 15].

Zur Diagnostik von *NTRK*-Genfusionen stehen verschiedene Verfahren zur Verfügung (Tab. 1). So lässt sich mithilfe der Immunhistochemie (IHC) die TRK-Expression bestimmen. Hierbei ist zu beachten, dass in bislang vorliegenden Studien dieses Verfahren mit monoklonalen TRK-Antikörpern getestet wurde. Den Antikörpern ist gemeinsam, dass sie ein C‑terminales Konsensusmotiv in TRKA‑C detektieren [1, 27, 42, 46]. Der zurzeit gebräuchlichste Antikörperklon ist EPR17341. Die TRK-Expression ist daher nicht beweisend für das Vorliegen einer *NTRK*-Genfusion, da auch die Expression des Wildtyp-TRK-Proteins nachgewiesen wird. Somit ist die Spezifität der IHC-Analyse eingeschränkt und kann zu falsch positiven Ergebnissen führen, beispielsweise in Tumoren abgeleitet von neuralem oder glattmuskulärem Gewebe mit physiologischer TRK-Expression. Auch für maligne klein-rund-und-blauzellige Tumoren des Kindesalters ist eine TRK-Expression beschrieben, ohne dass eine Fusion zugrunde liegt [57]. Während eine Untersuchung von Hirntumoren auf *NTRK*-Genfusionen mithilfe der IHC aufgrund der dargestellten Konstellation nicht sinnvoll möglich ist, bietet sich das Verfahren als vergleichsweise rasch und einfach etablierbare Screeningmethode für andere Tumorentitäten dennoch an. Solomon und Kollegen berichten in ihrer Arbeit, die über 38.000 per NGS analysierte Gewebeproben untersucht hat, über eine Sensitivität der NTRK-IHC von 96,2 % für *NTRK1*-Fusionen, 100 % für *NTRK2*-Fusionen und 79,4 % für *NTRK3*-Fusionen, wenn alle untersuchten Tumorentitäten kumuliert betrachtet wurden [57]. Innerhalb der untersuchten Tumorentitäten traten teils erhebliche Unterschiede zutage: Während beispielsweise für das Kolonkarzinom eine Sensitivität von 87,5 % und eine Spezifität von 100 % beobachtet wurden, lag diese für Mammakarzinome bei 80 % bzw. 82,1 % und für Speicheldrüsentumoren bei 88,9 % bzw. 52 %. Hingegen wurde für den inflammatorischen myofibroblastären Tumor eine Sensitivität und Spezifität von jeweils 100 % gemessen. Weitere Daten großer Fallkollektive, die in unabhängigen Studien erhoben wurden, sind notwendig, um die Kennwerte der IHC abschließend bewerten zu können. Zusammenfassend ist jedoch festzuhalten, dass die bisher erhobenen Daten zeigen, dass sowohl die Spezifität als auch die Sensitivität vom analysierten Gewebetyp und der jeweiligen *NTRK*-Genfusion beeinflusst wird und nicht immer 100 % erreicht [57], sodass in einem IHC-basierten Screeningverfahren neben falsch positiven auch falsch negative Befunde generiert werden können, dies gilt vor allem für *NTRK3*-Fusionen [20, 24].

**Table 1:** 

	IHC	FISH	RT-PCR	NGS
*Untersuchungsmaterial*	Formalinfixiertes paraffineingebettetes (FFPE) Gewebe	Frisches oder FFPE-Gewebe	FFPE-Gewebe, schockgefrorenes Gewebe	FFPE-Gewebe, frisches, gefrorenes, schockgefrorenes Gewebe
*Turnaroundzeit* (Zeit vom Eingang des Materials bis zum Befund)	Ergebnis in 1–2 Tagen verfügbar	Ergebnis in 2 Tagen verfügbar	Ergebnis in 5–10 Tagen verfügbar	Ergebnis nach 2 Wochen verfügbar
*Vorteile*	Schnell und kostengünstig	Weit verbreitete und etablierte Methode	Kostengünstiges und relativ schnelles Verfahren	Kann neue *NTRK*-Fusionspartner identifizieren
	Etabliertes Verfahren, weit verbreitet	Schnell	Zuverlässige Methode, um bekannte und wiederholt auftretende Genfusionen zu erkennen, z. B. *ETV6-NTRK3*	Gewebesparende Methode
	Wenig Material (Gewebe) erforderlich	Verlässliches Verfahren, um bekannte und wiederholt auftretende Genfusionen zu erkennen, z. B. *ETV6-NTRK3*	3′/5′-*NTRK*-Ratio: Ungleichgewicht zugunsten der 3′-Region als möglicher Hinweis auf das Vorliegen einer Genfusion	Evaluierung diverser Zielstrukturen in einer Untersuchung
	Relativ hohe Sensitivität (95–100 %, 75–96 %) und Spezifität (92/93–100 %)	Prinzipiell zum Nachweis auf das Vorliegen von *NTRK1-, NTRK2-* oder *NTRK3-*Genfusionen geeignet		
	→ Aber: Bislang nur in kleinen Patientengruppen getestet			
*Nachteile*	Nicht spezifisch für *NTRK*-Genfusionen, da sowohl Wildtyp- als auch Fusionsproteine detektiert werden	3 verschiedene Assays erforderlich, um *NTRK1-, NTRK2-* und *NTRK3-*Genfusionen zu erkennen	Zielsequenz muss bekannt sein, unbekannte oder neue Partner (5′-Region) werden nicht ohne Weiteres entdeckt	Eventuell Identifizierung aller *NTRK*-Genfusionen mit kommerziellen DNA-basierten Plattformen nicht möglich, vor allem bei *NTRK2* und *NTRK3*-Genfusionen, da viele Introns vorhanden
	Ggf. schwierige Auswertung in Geweben mit physiologischer TRK-Expression	Keine Identifizierung des Fusionspartners möglich, sofern dieser nicht bekannt ist	3′/5′-*NTRK*-Ratio: Sensitivität abhängig von Unterschied in der Expression des Wildtypgens und der Genfusion (nicht ausreichend untersucht)	RNA-basierte Tests stark von der Qualität der RNA abhängig
	Vor allem TRKC (*NTRK3*-Genfusionen) schwierig zu diagnostizieren	Nicht bekannt, ob das entsprechende Produkt der Genfusion exprimiert wird		Ergebnisse erst nach 2 Wochen bekannt
	Keine Identifizierung des Fusionspartners möglich			
	Keine Anwendung bei ZNS-Tumoren			

*FFPE* formalinfixiert und paraffineingebettet, *FISH* Fluoreszenz-in-situ-Hybridisierung, *IHC* Immunhistochemie, *NGS* Next Generation Sequencing, *RT-PCR* Reverse-Transkriptase-Polymerase-Kettenreaktion, *ZNS* zentrales Nervensystem

Bei der FISH-Untersuchung handelt es sich um eine weit verbreitete Methode zur Bestimmung chromosomaler Translokationen [1, 10, 27, 42, 46, 56]. Sie erkennt bei der Verwendung von sog. Break-apart- oder Fusionssonden den chromosomalen Bruch eines Gens, nicht aber die Genfusion selbst inklusive des Fusionspartners. Um bekannte und wiederholt auftretende Genfusionen zu diagnostizieren, gilt die FISH generell als robustes und verlässliches Verfahren mit hoher Spezifität. Allerdings zeigen *NTRK*-Genfusionen mitunter nichtkanonische Bruchpunkte, die aufgrund des Designs der jeweiligen Hybridisierungsproben nicht erkannt werden und somit zu einem falsch negativen Befundergebnis führen können. Ferner wird mit der Methode nicht festgestellt, ob das resultierende Genprodukt transkribiert oder exprimiert wird.

Auch die RT-PCR gilt als kostengünstiges und relativ schnelles Verfahren [1, 10, 27, 42, 46, 56, 57]. Mit diesem Verfahren lassen sich bekannte und wiederholt auftretende Genfusionen auf Transkriptebene zuverlässig erkennen. Aufgrund des großen Spektrums an *NTRK-*Genfusionen, die seltene Fusionspartner und variable Exone involvieren, ist das Verfahren technisch jedoch nicht geeignet, um eine umfassende Fusionsanalytik durchzuführen. Insbesondere können neue Genfusionen, die neue Fusionspartner und unterschiedliche Exone umfassen, nicht identifiziert werden.

Nach aktuellem Stand der Literatur ist das fokussierte, panelbasierte NGS – neben der FISH – das am besten geeignete Verfahren, um *NTRK*-Genfusionen in formalinfixiertem und paraffineingebettetem Probengewebe zu identifizieren [10]. Es lassen sich 2 Methoden unterscheiden: das RNA- und das DNA-basierte NGS [1, 10, 27, 42, 46, 56, 57]. Je nach eingesetztem Verfahren können damit auch neue *NTRK*-Fusionspartner bzw. Genfusionen entdeckt werden. Die Menge der für die Analytik verwendeten Nukleinsäuren ist assayabhängig und muss im Hinblick auf das Probenmaterial beachtet werden. Der Analysezeitraum („turnaround time“, TAT) dieser Verfahren ist abhängig von den Ressourcen und Kapazitäten der Labore sowie eingesetzten Methoden und Technologien. Die Analyse dauert aber in der Regel länger als eine IHC- oder FISH-basierte Untersuchung. Es ist wichtig festzuhalten, dass es sich sowohl bei der DNA- als auch bei der RNA-basierten NGS-Panel-Analyse um eine selektive Analyse handelt und nicht um eine Ganzgenomanalyse oder ein vollständiges Transkriptom. Dies ist relevant, da die Bruchpunkte variabel in intergenischen/intronischen Bereichen vorkommen, die in keinem fokussierten DNA-Assay vollständig enthalten sind. Ferner können diese Bruchpunkte in teils schwierig sequenzierbaren Regionen gelegen sein (z. B. in GC-reichen Regionen). Daher kann ein DNA-Assay falsch negative Befunde erzeugen [6, 56, 57]. Solomon et al. berichten beispielsweise, dass mit dem DNA-basierten MSK-IMPACT-Panel für *NTRK1*-, *NTRK2*- und *NTRK3*-Genfusionen eine Sensitivität von 96,8 %, 0 % bzw. 76,9 % gemessen wurde [57]. Die Sensitivität des Assays für alle *NTRK*-Genfusionen lag integral bei 81,1 %. Wahr negative Fälle wurden fast vollständig als negativ erkannt (99,86 % Spezifität). Die Kollegen beschreiben in der Arbeit, dass die meisten Fälle, die mithilfe der DNA-Analytik nicht erkannt wurden, über ein RNA-NGS-Verfahren identifiziert wurden. In einer anderen Studie der gleichen Institution wurden mehr als 2500 Adenokarzinome der Lunge mittels DNA-basierter NGS-Analytik untersucht und dann der Zusatznutzen eines RNA-basierten Assays analysiert [6]: In 32 % der per DNA-Sequenzierung als treibernegativ erkannten Tumoren, die darüber hinaus eine eher niedrige Mutationslast aufwiesen, konnte etwa bei einem Drittel dieser Fälle in der RNA-Analytik nachträglich ein therapierbarer Treiber (Genfusion oder Met-Exon14-Skipping) identifiziert werden, darunter auch 2 Fälle mit einer *NTRK3*-Genfusion. Es ist aber zu beachten, dass auch die fokussierte Untersuchung der RNA, die nicht in der Lage ist, den exakten Bruchpunkt auf DNA-Ebene zu detektieren, wohl aber das Transkript des fusionierten Gens, mit Limitationen behaftet ist. Je nach Design des Assays werden nicht alle Genfusionen detektiert, da z. B. Primer nur bestimmte, aber nicht alle möglichen codierenden Exone erkennen, die für das jeweilige Transkript codieren [31, 47, 56, 57]. Es ist auch zu berücksichtigen, dass die RNA labiler als DNA ist und die derzeit verfügbaren Assays zur RNA-basierten Fusionsanalytik unterschiedliche RNA-Mengen benötigen [31, 65].

### Vorhandene Untersuchungsalgorithmen

Mit dem bisherigen Wissensstand bietet es sich an, einen Diagnosealgorithmus zu entwickeln. Hierbei sind jedoch verschiedene Herausforderungen zu bewältigen. Dazu gehören etwa die relative Seltenheit der TRK-Fusionstumoren sowie die Vielzahl der potenziell betroffenen Tumorentitäten. Nicht zuletzt sind die Kosten zu berücksichtigen.

In verschiedenen Publikationen wurden Algorithmen zur Diagnose von TRK-Fusionsproteinen bzw. *NTRK*-Genfusionen vorgestellt und diskutiert. So veröffentlichte die European Society for Medical Oncology (ESMO) 2019 Empfehlungen zur Diagnose von *NTRK*-Genfusionen und der TRK-(Über‑)Expression [42]. Weitere Algorithmen zur Diagnose stellten Wong et al. [67], Penault-Llorca et al. [46], Hsiao et al. [27], Pfarr et al. [47] und Naito et al. [43] vor.

### Erarbeitung eines Untersuchungsalgorithmus

Wir schlagen einen modifizierten Algorithmus vor, der die unterschiedlichen Wahrscheinlichkeiten für das Vorliegen einer *NTRK*-Genfusion und auch die entstehenden Kosten berücksichtigt. Zudem wird einbezogen, dass Tumoren, bei denen *NTRK*-Genfusionen häufig vorkommen, wie das IFS oft mit anderen Therapien erfolgreich behandelt werden können.

Bei positivem Ergebnis des IHC-Screenings auf TRK-Fusionstumoren müssen bestätigende Tests auf genomischer Ebene mit einer FISH/ISH oder NGS erfolgen. Mögliche Vor- und Nachteile dieser Untersuchungen sind in Tab. 1 aufgeführt.

**Figure 2:**
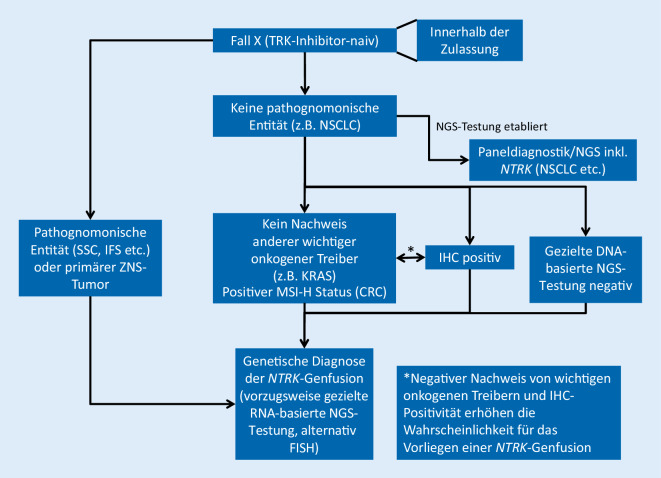

Der hier präsentierte Vorschlag (Abb. 2) für einen Diagnosealgorithmus berücksichtigt die genannten Herausforderungen. Bei Tumoren mit hoher Wahrscheinlichkeit für *NTRK*-Genfusionen, z. B. sekretorisches Speicheldrüsen- und Mammakarzinom, IFS etc. sowie bei primären Tumoren des Zentralnervensystems (ZNS) wird eine direkte molekulare Analytik empfohlen und die IHC als Zwischenschritt nicht durchgeführt. Bei Tumoren mit geringerer Wahrscheinlichkeit sind 2 Szenarien zu unterscheiden: Zum einen gibt es Tumorentitäten, bei denen eine NGS-basierte Fusionsanalyse bereits durchgeführt wird, z. B. beim NSCLC. Liegt eine solche Konstellation vor, ist ein vorgeschaltetes IHC-Screening überflüssig. Sollte die NGS-Analyse des NSCLC Genfusionen nicht erfassen, kommt ein sequenzielles Vorgehen mithilfe der IHC und FISH oder direkt mit einer FISH-Analyse in Betracht. Umfassende Vergleichsdatensätze liegen noch nicht vor, aber die Daten von Solomon et al. legen nahe, dass die tatsächlich negativen Fälle mit Hilfe der IHC durchweg als negativ identifiziert werden (Spezifität: 100 %), während die Sensitivität der IHC in der Studie nur bei ca. 87 % lag, sodass positive Fälle möglicherweise in der IHC-Analyse nicht erkannt werden [57]. Diagnostisch hilfreich ist in dem Kontext auch das Konzept des einander ausschließenden Treiberveränderungen: Ein NSCLC ohne Nachweis einer typischen Treiberveränderung (EGFR, ALK, ROS, RET, BRAF, KRAS etc.) erhöht die Prätestwahrscheinlichkeit für die Detektion eines anderen Treibers inklusive der *NTRK*-Genfusionen.

Zum anderen gibt es zahlreiche Tumorentitäten, bei denen eine umfassende Genfusionsanalytik derzeit kein Standard ist. Hier sollten aus ökonomischen Gesichtspunkten In-situ-Verfahren wie die IHC (mit oben genannten Einschränkungen) oder FISH zur Vorselektion berücksichtigt werden. Für die Fallauswahl sollten neben dem Stadium und den noch zur Verfügung stehenden klinischen Therapiemöglichkeiten auch z. B. der fehlende Nachweis anderer typischer Treibermutationen (z. B. duktales Adenokarzinom mit *KRAS*-Wildtyp-Status, 4fach negativer GIST [gastrointestinaler Stromatumor]) berücksichtigt werden. Kürzlich publizierte Daten legen nahe, dass Kolonkarzinome mit MSI-H-Status und fehlendem Nachweis klassischer onkogener Treiber vermehrt *NTRK*-Fusionen aufweisen [7, 48]. Es ist zu erwarten, dass sich mit wachsendem wissenschaftlichem Erkenntnisgewinn im Laufe der Zeit das Spektrum molekularer Profile, die möglicherweise helfen, *NTRK*-positive Tumoren zu identifizieren, erweitert. Klinische Parameter, wie z. B. junge Patienten, keine regelmäßige Exposition zu typischen externen Noxen, können ebenfalls hilfreich sein. Auch hier ist zu erwarten, dass der wissenschaftliche Kenntnisstand wachsen wird. Grundsätzlich muss ein positives IHC-Ergebnis nachfolgend durch ein anderes Nachweisverfahren, das die zugrunde liegende Translokation/Genfusion erkennt (z. B. FISH oder NGS) bestätigt werden. Das Ergebnis der IHC allein ist aufgrund der weiter oben dargestellten Konstellation nicht hinreichend.

Zu beachten ist auch, dass, wie in Abb. 2 dargestellt, aufgrund der weiter oben dargestellten Konstellation ein negatives *NTRK*-Fusionsergebnis mittels eines DNA-basierten NGS-Panels auch falsch negativ sein kann, sodass bei entsprechender Plausibilitätsanalyse (beispielsweise junger Patient ohne detektierten Treiber auf DNA-Ebene) eine ergänzende RNA-Analyse zur Fusionsdetektion erwogen werden kann [6].

Für die pädiatrische Onkologie ist eine zentrale referenzpathologische Diagnostik im Rahmen der verschiedenen Therapieoptimierungsstudien solider Tumoren der Gesellschaft für Pädiatrische Onkologie und Hämatologie (GPOH) etabliert. In diesem Rahmen ist auch eine panelbasierte NGS-Analytik für pädiatrische Sarkome aufgebaut worden. Die INFORM-Plattform bietet für Hochrisikoerkrankungen im Rezidivfall, aber auch für bestimmte Hochrisikoerkrankungen im Rahmen der Primärdiagnose (z. B. metastasierte Rhabdomyosarkome und hochmaligne Gliome [HGG]) umfassende molekulare Analysen an (WES, lcWGS, RNAseq und DNA-Methylierung) [63]. Für niedrigmaligne Gliome (LGG) steht bundesweit die LOGGIC-Core-Plattform zur Verfügung, die eine panelbasierte NGS-Analytik und eine RNA-Sequenzierung in Fällen anbietet, bei denen die Treiberalteration mittels Panel nicht identifiziert wurde [30, 40, 41, 58].

### Klinische Testsituation und Testzeiträume

Für die Testung sollte möglichst aktuelles Gewebe verwendet werden. Wenn dies nicht der Fall ist, ist der Rückgriff auf älteres Archivmaterial möglich, da eine *NTRK*-Genfusion in der Regel eine sog. Stammmutation („truncal mutation“) ist, die früh im Rahmen der Tumorevolution auftritt und tumortreibend ist. Eine Ausnahme sind sekundäre *NTRK*-Genfusionen, die als Resistenzmechanismus im Rahmen einer Tyrosinkinaseinhibitortherapie beschrieben worden sind [70]. Der Testzeitpunkt muss sich an den aktuellen, in den entitätsspezifischen Leitlinien festgelegten, stadienabhängigen Therapieverfahren orientieren und den Zulassungstext im Hinblick auf den zu untersuchenden klinischen Fall berücksichtigen [5, 13, 21]:

1. lokal fortgeschrittene, metastasierte Tumoren oder Tumoren, bei denen chirurgische Resektionen mutmaßlich zu einer hohen Morbidität führen
  *oder*
2. es keine zufriedenstellenden Therapieoptionen gibt.

Die Zulassung erstreckt sich auf alle soliden Tumorentitäten.

Derzeit gibt es keine internationalen Empfehlungen oder Leitlinien, die die Turnaroundzeiten (TAT), definiert als Zeitraum zwischen Erhalt des Gewebes bis zur Versendung des Befundes, für die molekulare Diagnostik in Tumorentitäten außer dem NSCLC abschließend festlegt. Die internationale Leitlinie zur molekularen Testung von Lungenkrebspatienten definiert einen Zeitraum von 10 Werktagen, der sicherlich einen Orientierungspunkt darstellt [38]. Aus Sicht der Autoren ist daher ein Testergebnis innerhalb von etwa 10 Werktagen anzustreben. Hierbei ist jedoch anzumerken, dass die Menge und Qualität des Gewebes und der Nukleinsäuren die TAT beeinflusst und im Einzelfall längere TAT möglich sein können.

## Wie können TRK-Fusionstumoren behandelt werden?

Tumoren mit *NTRK*-Genfusionen bzw. TRK-Fusionstumoren können seit kurzem mit TRK-Inhibitoren behandelt werden. Derzeit liegt für einen TRK-Inhibitor (Larotrectinib) eine Zulassung in der Europäischen Union vor (s. Abschn. „Larotrectinib“). TRK-Inhibitoren sind für die Therapie von Tumoren mit somatischen *NTRK*-Punktmutationen, *NTRK1*-Splicevarianten oder einer TRK-Überexpression des Wildtypproteins nicht zugelassen bzw. geeignet. Zur Beurteilung der Wirksamkeit von TRK-Inhibitoren wie Larotrectinib bei *NTRK*-Mutationen und *NTRK*-Genamplifikationen liegen bislang keine ausreichenden Daten vor, die einen therapeutischen Einsatz rechtfertigen würden [25].

### TRK-Inhibitoren

TRK-Inhibitoren sind Tyrosinkinaseinhibitoren aus der Klasse der „small molecules“. Derzeit befinden sich mehrere in der Entwicklung bzw. sind bereits zugelassen. Dazu gehört Larotrectinib, ein hochselektiver und hochpotenter Inhibitor aller 3 TRK-Proteine (Pan-TRK-Inhibitor), der praktisch keine Mitglieder anderer Kinasefamilien hemmt [15]. Ein weiterer Vertreter ist Entrectinib, ein Pan-TRK-Inhibitor, der auch Fusionsproteine von ROS1 (Protoonkogen-Protein-Tyrosinkinase) und ALK inhibiert [10]. Weiterhin hemmt Entrectinib Januskinase 2 und TNK2 („tyrosine kinase non receptor 2“) [21]. Die Zulassung wurde als TRK- und ROS1-Inhibitor beantragt. Weitere TRK-Inhibitoren sind Selitrectinib und Repotrectinib, die sich gegenwärtig in der klinischen Prüfung befinden. Ersterer ist ein TRK-Inhibitor der nächsten Generation, der bei Resistenzmutationen gegenüber Larotrectinib oder Entrectinib eingesetzt werden kann [10]. Letzterer gilt als TRK-, ROS1- und ALK-Inhibitor der zweiten Generation, der bei Resistenzmutationen gegenüber Entrectinib (bei TRK- oder ROS1-Resistenz) oder Larotrectinib (bei TRK-Resistenz) wirksam sein kann [10].

### Wirksamkeit von TRK-Inhibitoren

#### Larotrectinib

Larotrectinib ist weltweit bereits in mehr als 30 Ländern zugelassen, darunter in den USA [54] und in der Europäischen Union (EU) [5]. Die Anwendung kann nach dem Zulassungstext als orale Monotherapie zur Behandlung von erwachsenen und pädiatrischen Patienten mit soliden Tumoren mit einer *NTRK*-Genfusion [5] erfolgen,

- bei denen eine lokal fortgeschrittene oder metastasierte Erkrankung vorliegt oder eine Erkrankung, bei der eine chirurgische Resektion wahrscheinlich zu schwerer Morbidität führt und
- für die keine zufriedenstellenden Therapieoptionen zur Verfügung stehen.

Die Zulassung für Larotrectinib in der EU beruht auf den Ergebnissen gepoolter Analysen [26, 29, 35] von Patienten mit TRK-Fusionstumoren aus einer Phase-1-Studie mit Erwachsenen [25], einer Phase-1/2-Studie mit Kindern und Heranwachsenden (≤21 Jahre; SCOUT) [33] und einer Phase-2-Studie mit Heranwachsenden (≥12 Jahre) und Erwachsenen (NAVIGATE), die mit Larotrectinib behandelt wurden. Die Patienten wiesen insgesamt 25 verschiedene Tumorentitäten auf: IFS, andere Sarkomentitäten, Melanome, Tumoren der Schilddrüse, der Lunge, des Dickdarms, der Speicheldrüsen sowie primäre ZNS-Tumoren. Etwa die Hälfte der Patienten hatte keine oder nur eine systemische Vortherapie erhalten, die andere Hälfte 2 oder mehr Vortherapien (Tab. S1). Die Beurteilung des Tumoransprechens erfolgte nach den RECIST-v1.1-Kriterien bzw. bei ZNS-Tumoren/Filiae nach RANO-Kriterien.

##### Wirksamkeit von Larotrectinib.

In der bislang letzten Auswertung lag die Altersspanne der Effektivitätskohorte (*n* = 159) zwischen <0,1 und 84 Jahren (Tab. S1; [26, 29]). Die aus den Dosisfindungsstudien resultierende Dosierung betrug 100 mg zweimal täglich für Erwachsene bzw. 100 mg/m^2^ Körperoberfläche (jedoch nicht mehr als 100 mg) zweimal täglich bei Kindern und Jugendlichen, die in Form von Kapseln oder einer Lösung eingenommen werden konnten. Die Prüfarzt-bestimmte objektive Ansprechrate (ORR, primärer Endpunkt) betrug 79 %, das mediane progressionsfreie Überleben (PFS) 28,3 Monate und das mediane Gesamtüberleben (OS) 44,4 Monate [26, 29]. Weitere Ergebnisse sind in der Tab. S2 zusammengefasst. Zum Zeitpunkt der Analyse setzten 74 % aller Patienten die Therapie fort.

Die Wirksamkeit von Larotrectinib bei primären oder sekundären TRK-Fusionstumoren des ZNS wurde ebenfalls berichtet [17, 26]. Eingeschlossen waren 18 Patienten mit primären Hirntumoren und 12 Patienten mit Hirnmetastasen. Unter den Patienten mit primären Hirntumoren hatten 15 (83 %) mindestens eine systemische Vortherapie erhalten, 13 waren zuvor operiert oder bestrahlt worden. In dieser Gruppe lag das mediane Alter bei 10 Jahren (Spanne 1 Jahr–79 Jahre). Die ORR betrug bei den 14 Patienten mit analysierbaren Daten zum Auswertungszeitpunkt 36 % (Tab. S2). Die Therapie wurde in den meisten Fällen zu diesem Zeitpunkt noch fortgeführt. Patienten mit Hirnmetastasen wiesen eine ORR von 75 % auf (Tab. S2).

#### Entrectinib

Entrectinib ist in den USA schon sowohl zur Therapie von Erwachsenen mit ROS1-positivem NSCLC als auch zur Therapie von erwachsenen und pädiatrischen Patienten ab 12 Jahren mit soliden Tumoren zugelassen, die [21]

- eine *NTRK*-Genfusion ohne eine erworbene Resistenzmutation aufweisen und
- metastasiert sind oder bei denen eine chirurgische Resektion wahrscheinlich zu schwerer Morbidität führt, und
- nach einer Therapie progredient waren oder für die keine zufriedenstellende alternative Therapie verfügbar ist.

Entrectinib steht als Kapsel zur Verfügung [21]. Die empfohlene Dosierung beträgt für Erwachsene einmal täglich 600 mg und für Heranwachsende ab 12 Jahren abhängig von der Körperoberfläche (KOF) einmal täglich 600 mg (KOF >1,5 m^2^), 500 mg (KOF 1,1–1,5 m^2^) oder 400 mg (KOF 0,91–1,10 m^2^).

Die Wirksamkeit und Verträglichkeit von Entrectinib wurde in verschiedenen Studien bei Kindern, Heranwachsenden und Erwachsenen mit TRK-Fusionstumoren sowie mit ROS1- und ALK-positivem NSCLC evaluiert. Dazu gehören die Phase-1-Studien ALKA-372-001 und STARTRK‑1, die Phase-2-Studie STARTRK‑2 und die Phase-1/1b-Studie STARTRK-NG [39].

##### Wirksamkeit von Entrectinib.

In eine gepoolte Analyse wurden insgesamt 54 erwachsene Patienten mit TRK-Fusionstumoren aus der ALKA-372-001- (*n* = 1), STARTRK-1- (*n* = 2) und STARTRK-2-Studie (*n* = 51) eingeschlossen [12, 14, 50, 55]. Die 3 häufigsten Tumorarten waren Sarkome (24 %), NSCLC (19 %) und das sekretorische Speicheldrüsenkarzinom (13 %) [12, 14]. Fast 40 % der Teilnehmer hatten keine und ca. 20 % nur eine systemische Vortherapie, weitere 40 % 2 oder mehr Vortherapien erhalten (Tab. S3). Das mediane Alter der Patienten mit TRK-Fusionstumoren betrug 57,5 Jahre (Tab. S3). Primäre Endpunkte der gepoolten Analyse waren die ORR und die Dauer des Ansprechens (DOR, beide unabhängig bestimmt per RECIST v1.1) [12]. Die ORR betrug in der Gesamtpopulation 59,3 %, das mediane PFS 11,2 Monate und das mediane OS 20,9 Monate [50]. Weitere Ergebnisse zu Wirksamkeitsparametern sind in der Tab. S4 zusammengefasst. Bei 12 Patienten mit TRK-Fusionstumoren waren zu Studienbeginn ZNS-Metastasen nachweisbar [50, 55]. Bei diesen Patienten betrug die ORR 58,3 % (Tab. S4).

Daten zur Verträglichkeit von Larotrectinib und Entrectinib haben wir im elektronischen Supplement (S-Infobox 1) dargestellt.

### TRK-Inhibitoren: Anwendung bei Kindern und Heranwachsenden

Sowohl für Larotrectinib als auch für Entrectinib liegen in separaten Auswertungen erhobene Daten zur Wirksamkeit und Verträglichkeit bei Kindern und Heranwachsenden vor. So gibt es eine Analyse zu Larotrectinib in der Therapie von 38 jungen Patienten (<18 Jahre) mit lokal fortgeschrittenen oder metastasierten TRK-Fusionstumoren. Patienten mit primären ZNS-Tumoren waren hier nicht eingeschlossen [62]. Das Alter der Betroffenen reichte von 0,1 bis 14 Jahren, im Median lag es bei 2,3 Jahren. Fast die Hälfte der Teilnehmer wies ein IFS (47 %) auf, weitere Krebserkrankungen waren andere Weichgewebesarkome (42 %), Schilddrüsenkarzinome (5 %), CMN (3 %) und Melanome (3 %). Vor Studienbeginn hatten 32 % keine, 53 % 1–2 und 16 % mindestens 3 systemische Vortherapien erhalten. Die durch den Prüfarzt bestimmte ORR betrug 94 %. Zum Zeitpunkt des Datenschnitts und der anschließenden Analyse (30.07.2018) waren die mediane DOR, das mediane PFS und das mediane OS noch nicht erreicht. Von den Patienten wurden 87 % (33 von 38) zum Auswertungszeitpunkt mit Larotrectinib weiterbehandelt oder konnten sich einer kurativen chirurgischen Therapie unterziehen.

Auftretende unerwünschte Ereignisse (UE) wie erhöhte ALT (43 %), Erbrechen (41 %), erhöhte Aspartat-Aminotransferase-Werte (AST, 39 %), Diarrhö (34 %) sowie Husten und Neutropenie (je 32 %) waren überwiegend von Grad 1 oder Grad 2 [62]. Zu den häufigsten Grad-3-UEs zählten Neutropenie (13 %) und Gewichtszunahme (9 %). Grad-4-UEs waren mit je 2 % eine reduzierte Neutrophilenzahl und Fieber.

In der STARTRK-NG-Studie wurde die Therapie mit Entrectinib – in den USA zugelassen ab 12 Jahren – bei Kindern und Heranwachsenden evaluiert [21, 49]. Eingeschlossen waren 29 Patienten im Alter von 0–20 Jahren (Median: 7 Jahre). Bei 13 Patienten mit hochgradigen Gliomen, Sarkomen, embryonalen ZNS-Tumoren, Neuroblastomen oder Melanomen ließen sich *NTRK*- (*n* = 7), ROS1- (*n* = 3) oder *ALK*-Genfusionen (*n* = 3) feststellen. Zum Zeitpunkt der Analyse (31.10.2018) lebten alle Patienten mit *NTRK*-Genfusions‑, ROS1- oder ALK-positiven Tumoren, von denen 75 % weiterhin Entrectinib erhielten. Eine komplette Remission zeigte in der Gruppe mit TRK-Fusionstumoren ein Patient und bei 4 Patienten wurde eine partielle Remission erzielt. Insgesamt war zum Auswertungszeitpunkt die mediane DOR noch nicht erreicht, sie lag zwischen 1,8 und 15,7 Monaten. Die häufigsten UEs aller Grade waren Anämie und erhöhte Kreatininkonzentration im Blut (je 41 %), erhöhte ALT, erhöhte AST und Übelkeit (je 35 %). Grad-3/4-UEs traten bei insgesamt 10 % der Patienten auf, wobei die Neutropenie mit 5 Fällen (17 %) am häufigsten vorkam.

Sicherheitsdaten unter Langzeitexposition von TRK-Inhibitoren liegen noch nicht vor, vor allem keine Daten zu spezifisch pädiatrischen unerwünschten Wirkungen, wie z. B. ein Einfluss auf das Körperwachstum, die Pubertät oder die neurokognitive Entwicklung.

Generell ist jedoch zu berücksichtigen, dass sowohl bei der CMN als auch beim IFS der chirurgische Eingriff die wesentliche Therapieoption darstellt [22, 23, 34]. Bei fortgeschrittenen Tumoren kann eine Chemotherapie mit einem relativ geringen Risiko für Spätfolgen sinnvoll sein [45]. Daher wird der Einsatz von TRK-Inhibitoren beim IFS in der Erstlinie außerhalb von Studien zurzeit nicht empfohlen.

### Beginn einer Therapie mit TRK-Inhibitoren

Generell sieht der Zulassungstext für Larotrectinib und der wahrscheinlich ähnlich lautende für Entrectinib den Einsatz der Medikamente in späteren Therapielinien vor. Jedoch hat die European Medicines Agency (EMA) in ihrem „European Public Assessment Report“ (EPAR) für Larotrectinib festgestellt, dass prinzipiell auch ein Einsatz in früheren Therapielinien möglich ist, die Voraussetzungen hierfür sind, dass keine Standardtherapien existieren, oder, selbst wenn Therapien empfohlen werden, diese keinen nachgewiesenen und relevanten klinischen Vorteil zeigen [18].

Das Ziel einer Behandlung in diesem Krankheitsstadium ist die Reduktion von Symptomen und die Verlängerung der Überlebenszeit bei möglichst guter Lebensqualität. Patienten, die der Indikation von Larotrectinib entsprechen, haben also keine anderen Therapieoptionen mehr, die ein positives Nutzen-Risiko-Verhältnis erwarten lassen oder haben eine Chance auf Heilung nur durch mutilierende Operationen.

Die Indikation kann auch gegeben sein, wenn etwa noch vorhandene Salvage-Therapien aufgrund von zu erwartenden Toxizitäten oder Komorbiditäten, auch im Hinblick auf eine adäquate Lebensqualität, nicht (mehr) durchführbar sind. Der individuellen Abwägung durch den behandelnden Arzt, ob es noch Therapieoptionen gibt, die in der Bewertung des Nutzen-Risiko-Verhältnisses besser abschneiden als durch den Einsatz von TRK-Inhibitoren nach vorhandener Studienlage zu erwarten ist, kommt somit die entscheidende Bedeutung zu. Für diese Abwägung sind insbesondere die bekannten Daten zur ORR, zum PFS und der Verträglichkeit fundamental.

Es existieren bislang keine Daten, die Klarheit im Hinblick auf die notwendige Therapiedauer mit TRK-Inhibitoren liefern. In den gegenwärtigen Studien erfolgte eine Therapie bis zum Progress (in einzelnen Fällen auch darüber hinaus), dem Abbruch der Studie durch den Patienten oder dem Auftreten einer dosislimitierenden Toxizität [5, 15, 26, 29]. Ob und wie lange eine Therapie über eine komplette Remission hinaus notwendig ist, ist gegenwärtig unklar, genauso wie die Fragestellung, wie oft ein erneuter Einsatz eines TRK-Inhibitors bei einem Tumorprogress nach vorangegangenem Absetzen der Therapie wirksam ist (sog. Rechallenge). Zusammenfassend kann festgehalten werden, dass die Therapiedauer unklar ist, vor dem Hintergrund und dem Verständnis anderer tyrosinkinasetherapeutischer Konzepte scheint aber derzeit i. d. R. eine Therapie bis zum Progress oder bis zur Therapieunverträglichkeit gerechtfertigt. Sicherlich ist dies besonders im pädiatrischen Setting individuell zu diskutieren.

### Entwicklung von Resistenzen gegenüber TRK-Inhibitoren

Es ist beschrieben, dass fusionspositive Tumoren unter Therapie mit TRK-Inhibitoren Resistenzen entwickeln können [16]. Zugrunde liegende Mechanismen können zum einen sog. On-target-Alterationen sein. Dabei handelt es sich um Mutationen oder Amplifikationen, die in der Genfusion vorkommen [53], u. a. Basensubstitutionen in der *NTRK*-Kinasedomäne (Gatekeeper-Mutationen) wie F589L in *TRKA*, F633L in *TRKB* und F617L in *TRKC*, Solvent-Front-Mutationen wie G595R in *TRKA*, G639R in *TRKB* und G623R in *TRKC* sowie x‑DFG-motif-Mutationen wie G667C in *TRKA*, G709C in *TRKB* und G696A in *TRKC* [10, 28]. Es können aber auch sog. Off-target-Alterationen zur Resistenzentwicklung beitragen, z. B. mittels Aktivierung anderer, durch die Substanzen nicht gehemmter Signalwege („Bypass-Signalweg“) [53]. Bei einer Aktivierung des MAPK-Signalwegs als Bypass-Signalweg weisen erste präklinische Daten darauf hin, dass eine Kombination aus einem MEK- und einem TRK-Inhibitor wirksam sein könnte [11].

Zweitgenerations-TRK-Inhibitoren sind selbst bei bestimmten On-target-Alterationen wirksam [16]. Ein Vertreter ist Selitrectinib (BAY 2731954), das oral verfügbar ist und in präklinischen Untersuchungen Aktivität gegen erworbene Solvent-Front‑, Gatekeeper- und x‑DFG-motif-Mutationen zeigte. Selitrectinib wird in einer Phase-I/II-Studie (NCT03215511, LOXO-EXT-17005) und in Single-Patient-Protokollen geprüft [28]. Insgesamt wurden 31 Patienten, darunter 7 pädiatrische Patienten, mit TRK-Fusionstumoren wie Sarkomen, GIST, Pankreastumoren, sekretorischen Speicheldrüsenkarzinomen oder Glioblastomen mit einem medianen Alter von 37 Jahren (Spanne 1,25–72 Jahre) behandelt. Alle Patienten waren entweder intolerant gegenüber oder progredient unter einer vorherigen TRK-Inhibitor-Therapie. Bei einer Dosierung von maximal 100 mg zweimal täglich erwies sich Selitrectinib als verträglich. Es zeigten sich erste Hinweise auf Wirksamkeit gegen Tumoren mit erworbenen Resistenzmutationen in der Kinasedomäne [16].

Ein weiterer Vertreter ist Repotrectinib (TPX-0005). Die Substanz wird derzeit in der Phase-I/II-Studie TRIDENT‑1 (NCT03093116) überprüft [10]. Eingeschlossen werden können u. a. Patienten mit fortgeschrittenen soliden Tumoren, inklusive primären ZNS-Tumoren, mit vorheriger TRK-Inhibitor-Therapie [9].

## Fazit für die Praxis

- *NTRK*-Genfusionen sind starke onkogene Treiber, die tumorentitätenübergreifend vorkommen.
- Die Wirksamkeit und Verträglichkeit der TRK-Inhibitoren Larotrectinib und Entrectinib wurden in Phase-I- und Phase-II-Studien gezeigt. Die bisherigen Daten legen nahe, dass eine Langzeitgabe möglich ist.
- Entrectinib erwies sich ebenfalls als wirksam bei TRK-Fusionstumoren, aber auch bei ROS1-positiven Tumoren. Die Substanz war ebenfalls gut verträglich und hatte ein gut zu beherrschendes Sicherheitsprofil.
- Es fehlen derzeit Informationen zu den möglichen Langzeitfolgen einer Therapie mit TRK-Inhibitoren.
- Die geringe Inzidenz der TRK-Fusionstumoren stellt die Diagnostik vor Herausforderungen. Zum einen sollten alle Patienten, die TRK-Fusionstumoren aufweisen, eine wirksame Therapie erhalten können, zum anderen muss der Diagnostikansatz die Epidemiologie, die jeweilige Tumorhistologie sowie die Ausstattung und Ressourcen des pathologischen Instituts berücksichtigen.
- Mit dem hier vorgestellten Diagnosealgorithmus möchten wir einen praktikablen und zuverlässigen Weg für die Diagnose von TRK-Fusionstumoren vorschlagen.

## Caption Electronic Supplementary Material


