# Supplementary material for: Diagnosis and therapy of tumors with NTRK gene fusion
Source: Pathologe. 2020 Nov 30;42(1):103–15. [Article in German] doi: 10.1007/s00292-020-00864-y (PMC7858552; doi:10.1007/s00292-020-00864-y)
Supplement: Supplementary file 1 [file 292_2020_864_MOESM1_ESM.pdf]

Supplement zum Beitrag

Diagnostik und Therapie von Tumoren mit NTRK-Genfusionen

Prof. Dr. Albrecht Stenzinger1, Dr. Cornelis van Tilburg2, et al

1 Allgemeine Pathologie und pathologische Anatomie, Pathologisches Institut, Universitätsklinikum Heidelberg

2 Hopp-Kindertumorzentrum Heidelberg (KiTZ), Deutsches Krebsforschungszentrum (DKFZ) und Universitätsklinikum Heidelberg

Ergänzende Inhalte sowie Tabellen zu Larotrectinib und Entrectinib [Verträglichkeit; Baseline-Charakteristika; Wirksamkeit von Entrectinib bei TRK-Fusionstumoren; Therapiebedingte unerwünschte Ereignisse] sowie zusätzliche Referenzen zu Abb. 1

S-Infobox 1: Verträglichkeit von Larotrectinib und Entrectinib

Larotrectinib

Zu den häufigsten unerwünschten Ereignissen (UEs, alle Grade) unter Larotrectinib zählten z.B. Fatigue, erhöhte Alanin-Aminotransferase (ALT) und Husten, in den meisten Fällen allerdings nur vom Schweregrad 1 oder 2 nach CTCAE (*common terminology criteria for adverse events*) (Tab. S5) [26, 29, 34]. UEs ≥ Grad 3 traten insgesamt selten auf (Tab. S5). Lediglich 2% (6 von 260 Patienten in der Sicherheitskohorte) der Patienten brachen die Therapie ab.

Entrectinib

In die Sicherheitspopulation waren 68 Patienten mit TRK-Fusionstumoren eingeschlossen worden [12, 14]. Die meisten UEs waren nach CTCAE Grad 1 oder 2 und reversibel. Am häufigsten traten Dysgeusie, Obstipation und Fatigue auf (Tab. S6). Grad-3-UEs umfassten u. a. Anämie, Gewichtszunahme und Fatigue. Das Sicherheitsprofil der Subgruppe mit TRK-Fusionstumoren entsprach dem der Gesamtpopulation (n=355) [14, 21]. Hier hatten aufgrund von UEs 3,9% der Patienten die Therapie abgebrochen.

Weiterhin fiel eine erhöhte Frakturrate auf: In einer erweiterten Sicherheitspopulation mit 338 erwachsenen und 30 pädiatrischen Patienten, die Entrectinib erhielten, kamen bei 5% bzw. 23% Frakturen vor [21]. Dabei traten die Frakturen bei den Erwachsenen in einigen Fällen im Zusammenhang mit Stürzen oder anderen Traumata auf. Inwieweit in der pädiatrischen Population den Frakturen Traumata vorausgingen, lässt sich aus den vorliegenden Daten nicht bewerten. Des Weiteren ließ sich eine Verlängerung der QT-Zeit unter Entrectinib beobachten. Daher sollten Patienten mit einem erhöhten Risiko für ein verlängertes oder bereits bei Therapiebeginn bestehendem verlängerten QT-Intervall regelmäßig untersucht werden.

Sowohl Entrectinib als auch Larotrectinib haben ein Arzneimittelinteraktionspotenzial, vor allem über CYP3A [5, 21].

Tab. S1: Baseline-Charakteristika in den gepoolten Analysen zu Larotrectinib		
	Datenschnitt: 30.07.2018 [34] n=122	Datenschnitt: 19.02.2019 [26, 29] n=159
**Geschlecht, n (%)**		
Männlich	60 (49)	77 (48)
Weiblich	62 (51)	82 (52)
**Medianes Alter, Jahre (Spanne)**	41 (0,1–80)	43 (< 0,1–84)
Kinder und Jugendliche (< 18 Jahre), n (%)		52 (33)
Erwachsene (≥ 18 Jahre), n (%)		107 (67)
< 2 Jahre	18 (15)	
2–< 6 Jahre	7 (6)	
6–< 15 Jahre	14 (11)	
15–< 39 Jahre	21 (17)	
≥ 40 Jahre	62 (51)	
**ECOG PS**		
0	57 (47)	76 (48)
1	53 (43)	61 (38)
2	12 (10)	19 (12)
3	0 (0)	3 (2)
**Anzahl systemischer Vortherapien, n (%)**		
0–1	66 (54)	83 (52)
2	25 (20)	34 (21)
≥ 3	31 (25)	42 (26)
**NTRK-Genfusionen, n (%)**		
NTRK1		64 (40)
NTRK2		4 (3)
NTRK3		88 (55)
Nicht bestätig		3 (2)
ECOG PS: Eastern Co-operative Oncology Group Performance Status		

Tab. S2: Wirksamkeit von Larotrectinib bei TRK-Fusionstumoren				
	Datenschnitt: 30.07.2018 [34] n=122	Datenschnitt: 19.02.2019 [26, 29] n=159	Datenschnitt: 19.02.2019 [26] Hirnmetastasen n=12	Datenschnitt: 19.02.2019 [17, 26] Primärer Hirntumor n=18
**Objektive Ansprechrate (ORR), % (95%-KI)**	81 (72–88) (n=109)	79 (72–85) (n=153)[b]	75	36
Komplette Remission (CR), %	17	16		14
Partielle Remission (PR), %	63	63	75	21
**Stabile Erkrankung (SD), %**	NR	12	17	64
**Progression (PD), %**	NR	6	8	0
**Zeit bis zum Ansprechen (median), Monate (Spanne)**	1,8	1,8 (0,9–6,1)	NR	NR
**Dauer des Ansprechens (median), Monate (95%-KI)**	NE (primäres und supplementäres Datenset[a])	35,2 (22,8–NE)[c]	NR	NR
**Progressionsfreies Überleben (median), Monate (95%-KI)**	NR	28,3 (22,1–nicht erreicht)[d]	NR	11,0 (2,8–nicht erreicht)
**Gesamtüberleben (median), Monate (95%-KI)**	NR	44,4 (36,5–nicht erreicht)[e]	NR	NR

[a]Medianes Follow-up für primäres Datenset: 17,6 Monate und für supplementäres Datenset 7,4 Monate, der angegebene Wert bezieht sich auf die Gesamtpopulation; [b]Bei 6 Patienten Ansprechen nicht bestimmt; [c]Medianes Follow-up: 12,9 Monate, bestimmt bei Patienten mit bestätigtem Ansprechen (n=108), der angegebene Wert bezieht sich auf die Gesamtpopulation; [d]Medianes Follow-up: 11,1 Monate, der angegebene Wert bezieht sich auf die Gesamtpopulation, im supplementären Datenset ist der Medianwert nach einem medianen Follow-up von 7,6 Monaten noch nicht erreicht; [e]Medianes Follow-up: 13,9 Monate, der angegebene Wert bezieht sich auf die Gesamtpopulation
NE: nicht erreicht, NR: nicht berichtet

Tab. S3: Baseline-Charakteristika in der Analyse zu Entrectinib	
	Datenschnitt: 31.05.2018 [12, 14]<br>*n=54*
**Geschlecht, %**	
Männlich	40,7
Weiblich	59,3
**Medianes Alter, Jahre (Spanne)**	57,5 (21–83)
**ECOG PS**	
0	42,6
1	46,3
2	11,1
**Anzahl systemischer Vortherapien, %**	
0	37,0
1	20,4
≥ 2	42,6

Tab. S4: Wirksamkeit von Entrectinib bei TRK-Fusionstumoren				
	Datenschnitt: 31.05.2018 [12, 14]	Datenschnitt: 30.10.2018 [47]		
	Alle Patienten n=54	Alle Patienten n=54	Mit ZNS-Metastasen n=12	Ohne ZNS-Metastasen n=42
**Objektive Ansprechrate (ORR), % (95%-KI)**	57	59,3 (45,0–72,4)	58,3 (27,7–84,4)	59,5 (43,3–74,4)
Komplette Remission (CR), %	7	7,4	0	9,5
Partielle Remission (PR), %	50	51,9	58,3	50,0
**Stabile Erkrankung (SD), %**	17	17,8	25,0	11,9
**Progression (PD), %**	7	7,4	0	9,5
**Keine komplette/partielle Remission**	6	5,6	0	7,1
**Keine Angaben/nicht evaluierbar**	13	13,0	16,7	11,9
**Dauer des Ansprechens (DOR, median), Monate (95%-KI)**	10,4 (7,1–NE)[a] (n=31)	12,9 (7,9–NE)	NE (4,2–NE)	12,9 (7,9–NE)
**Progressionsfreies Überleben (PFS, median), Monate (95%-KI)**	11,2 ((8,0–14,9)[b]	11,8 (8,0– 15,7)	9,7 (4,7–NE)	12,0 (8,3–16,0)
**Gesamtüberleben (OS, median), Monate (95%-KI)**	20,9 (14,9–NE)[b]	23,9 (16,8–NE)	–	–

a Medianes Follow-up: 13,1 Monate; **b** Medianes Follow-up: 12,9 Monate
NE: nicht erreicht

Tab. S5: Therapiebedingte unerwünschte Ereignisse von Larotrectinib		
	Datenschnitt: 30.07.2018 [34] n=207	Datenschnitt: 19.02.2019 [26, 29] n=260
**Alle Grade, % (≥ 10% der Patienten betroffen)**		
Erhöhte ALT	21	22
Erhöhte AST	19	20
Schwindel	21	18
Fatigue	18	17
Übelkeit	15	13
Obstipation	12	11
Anämie	11	10
Erbrechen	10	9
**≥ Grad 3, %**		
Erhöhte ALT	3	4
Erniedrigte Neutrophilenzahl		3
Anämie	2	2
Erhöhte AST	1	< 1
Erniedrigte Lymphozytenzahl		< 1
Fatigue	< 1	< 1
Kopfschmerzen		< 1
Myalgie	< 1	< 1
Schwindel	< 1	< 1
Übelkeit	1	< 1
ALT: Alanin-Aminotransferase, AST: Aspartat-Aminotransferase		

Tab. S6: Therapiebedingte unerwünschte Ereignisse unter Entrectinib (alle Patienten, die mit Entrectinib behandelt wurden)	
	n=355 [14]
**Alle Grade, % (≥ 10% der Patienten betroffen)**	
Dysgeusie	42
Fatigue	28
Schwindel	26
Obstipation	24
Diarrhö	22
Übelkeit	21
Gewichtszunahme	19
Parästhesie	19
Kreatinin erhöht	16
Myalgie	16
Anämie	15
Periphere Ödeme	15
Erbrechen	14
Arthralgie	13
Erhöhte AST	12
Erhöhte ALT	11
**≥ Grad 3, % (≥ 2% der Patienten betroffen)**	
Anämie	5
Gewichtszunahme	5
Fatigue	3
Neutropenie	3
Veringerte Neutrophilenzahl	2
ALT: Alanin-Aminotransferase, AST: Aspartat-Aminotransferase	

[S-Infobox 2] : Abb. 1: zusätzliche Referenzen

1. Ferguson SD et al. J Neuropathol Exp Neurol. 2018;77:437–442
2. Jones DT et al. Nat Genet. 2013;45:927–932
3. Qaddoumi et al. Acta Neuropathol. 2016;131:833–845
4. Yoshihara K et al. Oncogene. 2015;34:4845–4854
5. Subramaniam DS et al. J Clin Oncol. 2017;35:2019
6. Stransky N et al. Nat Comms. 2014;5:4846
7. Zheng Z et al. Nat Med. 2014;20:1479–1484
8. Kim J et al. PLoS One. 2014;9:e91940
9. Frattini V et al. Nat Genet. 2013;45:1141–1149
10. Serrano-Arevalo ML et al. Med Oral Pathol Cir Bucal. 2015;20:e23–e29
11. Krings G et al. Mod Pathol. 2017;30:1086–1099
12. Skálová A et al. Am J Surg Pathol. 2010;34:599–608
13. Ito Y et al. Am J Surg Pathol. 2015;39:602–610
14. Bishop JA et al. Hum Pathol. 2013;44:1982–1988
15. Skálová A et al. Am J Surg Pathol. 2016;40:3–13
16. Church AJ, et al. Mod Pathol. 2018;31:463–473
17. Guilmette J et al. Hum Pathol. 2019;83:50–58
18. Tognon C et al. Cancer Cell. 2002;2:367–376
19. Diallo R et al. Verh Dtsch Ges Pathol. 2003;87:193–203
20. Osako T et al. Histopathology. 2013;63:509–519
21. Lowery MA et al. Clin Can Res. 2017;23:6094–6100
22. Pishvaian MJ et al. Clin Can Res. 2018;24:5018–5027
23. Singhi AD et al. J Clin Oncol. 2018;36:292
24. Ross JS et al. Oncologist. 2014;19:235–242
25. Ling Q et al. Ann Oncol. 2018;29:mdy269.073
26. Lowery MA et al. Clin Can Res. 2018;24:4154–4161
27. Westphalen CB et al. Clin Transl Oncol. 2019;21(8):1108–1111
28. Bounacer A et al. Br J Can. 2000;82:308–314
29. Pfeifer A et al. Genes Chromosomes Cancer 2019;58(8):558–566
30. Brzezianska E et al. Mutat Res. 2006;599:26–35
31. Wajjwalku W et al. Jpn J Can Res. 1992;83:671–675
32. Bongarzone I et al. Clin Can Res. 1998;4:223–228
33. Bongarzone I et al. J Clin Endocrinol Metab. 1996;81:2006–2009
34. Sassolas G et al. Thyroid. 2012;22:17–26
35. Musholt TJ et al. Surgery. 2000;128:984–993
36. Leeman-Neill RJ et al. Cancer. 2014;120:799–807
37. Bastos AU et al. Eur J Endocrinol. 2018;178:83–91
38. Liu RT et al. Clin Endocrinol. 2005;63:461–466
39. Kitamura Y et al. J Hum Genet. 1999;44:96
40. Lee MY et al. Cancer Res Treat. 2017;49:906–914
41. Lee SE et al. Thyroid. 2017;27(6):802–810
42. Lu Z et al. Oncotarget. 2017;8(28):45784–45792
43. Park K et al. USCAP Meeting. 2018;98:235
44. Pesenti C et al. Endocrine. 2018;61:36–41

45. Sánchez Güerri SM et al. ECC. 2018;29:73
46. Cancer Genome Atlas Research Network. Cell. 2014;159:676–690
47. Smallridge RC et al. J Clin Endocrinol Metab. 2014;99:E338–E347
48. Costa V et al. Oncotarget. 2015;6:11242–11251
49. Beimfohr C et al. Int J Cancer. 1999;80:842–847
50. Benayed R et al. J Clin Oncol. 2018;36:12076
51. Nakamura A et al. Ann Oncol. 2017;28:mdx671.004
52. Wang W et al. J Thorac Oncol. 2018;13:S719
53. Farago AF et al. JCO Precis Oncol. 2018:2018
54. Varella-Garcia M et al. J Thorac Oncol. 2015;10:S246
55. Chen MW et al. Ann Oncol. 2017;28:vii16
56. Blakely CM et al. J Clin Oncol. 2016;34:e23079
57. Ou S et al. IASLC. 2017;12:S1848
58. Schrock AB et al. J Thorac Oncol. 2018;13:1312–1323
59. Suh JH et al. Oncologist. 2016;21:684–691
60. Brenca M et al. J Pathol. 2016;238:543–549
61. Shi E et al. J Transl Med. 2016;14:339
62. Park DY et al. Oncotarget. 2016;7:8399–8412
63. Créancier L et al. Cancer Letters. 2015;365:107–111
64. Hechtman JF et al. Mol Cancer Res. 2016;14:296–301
65. Lezcano C et al. Am J Surg Pathol. 2018;42:1052–1058
66. Boddu S et al. JCO Precis Oncol. 2018:1–8
67. Ravi V et al. Cancer Res. 2017;77:P2-12-01
68. Tauziede A et al. Virchows Archiv. 2018;473:PS-16-009
69. Wu G et al. Mod Pathol. 2016;29:359–369
70. Wang L et al. J Mol Diagn. 2017;19:387–396
71. Prasad ML et al. Cancer. 2016;122:1097–1107
72. Cordioli MI et al. Thyroid. 2017;27:182–188
73. Ricarte-Filho JC et al. J Clin Invest. 2013;123:4935–4944
74. Potter SL et al. Cancer Research. 2018;78:A36
75. Picarsic JL et al. Pediatr Dev Pathol. 2016;19:115–122
76. Rubin BP et al. Am J Pathol. 1998;153:1451–1458
77. Bourgeois JM et al. Am J Surg Pathol. 2000;24:937–946
78. Hung YP et al. Histopathology. 2018;73:634–644
79. Argani P et al. Mod Pathol. 2000;13:29–36
80. Knezevich SR et al. Cancer Research. 1998;58:5046–5048
81. El Demellawy D et al. Pathology. 2016;48:47–50
82. Pavlick D et al. Pediatr Blood Cancer. 2017;64:e26433
83. Cai J et al. Int J Cancer. 2019;144:117–124
